# Parasite transmission risk at the urban human–dog–macaque interface

**DOI:** 10.1017/S0031182026102339

**Published:** 2026-07

**Authors:** Anisha K C, Rosary Karki, Stefano S. K. Kaburu, Sabina Luitel, Kishor Pandey, Jenny Claire Dunn, Laxman Khanal, Laëtitia Maréchal

**Affiliations:** 1 Central Department of Zoology, Tribhuvan University, Kathmandu, Nepal; 2 School of Animal, Rural and Environmental Sciences, Nottingham Trent University, Southwell, UK; 3 School of Life Sciences, Huxley Building, Keele University, Newcastle-under-Lyme, UK; 4 International Centre for Biodiversity and Primate Conservation, Dali University, Dali, China; 5 School of Psychology, Sport Science and Wellbeing, College of Health and Sciences, University of Lincoln, Lincoln, UK

**Keywords:** human–dog–wildlife interface, parasite transmission, tourism and public health, urban ecology, zoonoses

## Abstract

Urbanization and tourism are intensifying human–animal interactions, yet their implications for cross-species parasite transmission remain poorly understood. This study investigates behavioural and ecological mechanisms underlying parasite transmission dynamics across interconnected human, dog and macaque populations sharing the same urban and touristic environment at Swayambhunath Temple, Kathmandu. We assessed parasite prevalence, diversity and infection intensity, combining parasitological analyses, participant questionnaire and behavioural observations to evaluate how anthropogenic factors and host behaviours influence parasitism and potential cross-species transmission. Humans displayed practices that may increase transmission risk, including close animal interactions, food provisioning and limited hygiene measures. Parasite prevalence was highest in macaques and dogs compared to humans, with macaques exhibiting the highest parasite diversity. Several parasites occurred in more than 1 species; notably, *Entamoeba* spp. was detected across all 3 species, indicating shared exposure pathways and zoonotic potential. In dogs, neither social nor anthropogenic variables predicted parasite metrics, but parasite diversity was linked to self-scratching, an indicator of anxiety, suggesting environmental pathway as a key drive of parasite transmission. In macaques, parasite diversity was shaped by interactions between dominance rank, provisioning and grooming, highlighting the interplay between social dynamics and human influence. Overall, these findings show that urban and touristic environments not only facilitate direct pathogen exposure but also indirectly shape parasite infection risk through behavioural and social mechanisms. Integrating behavioural ecology with urban disease ecology is essential for understanding and managing zoonotic risks in densely shared human–domestic animal–wildlife systems, with important implications for public and animal health and urban biodiversity management.

## Graphical abstract

**Figure:**
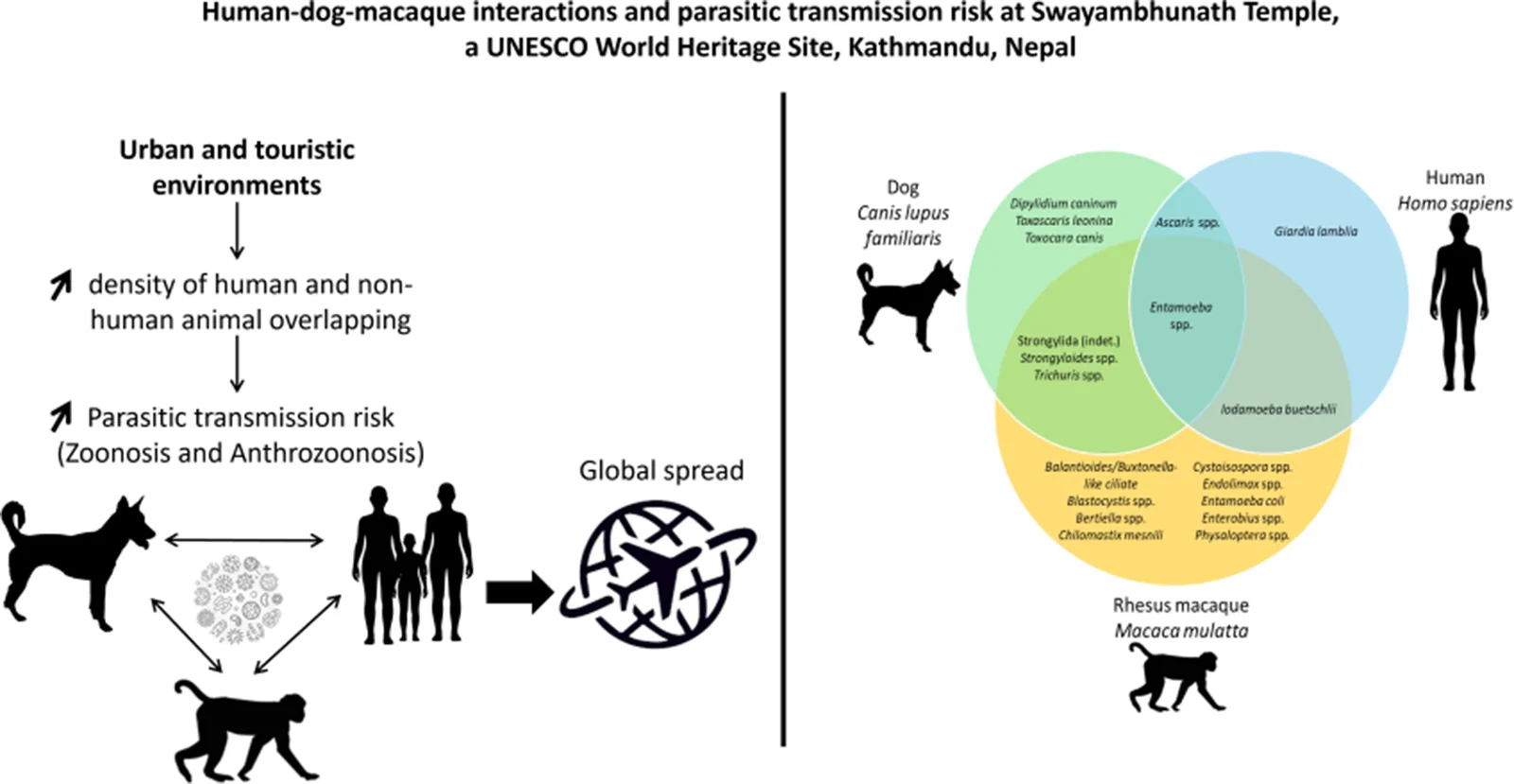

## Introduction

Emerging infectious diseases, approximately 70% of which originate in wildlife, represent a growing challenge for global public health (Bengis et al., 2004) and for animals living in anthropogenically dominated habitats. Rapid urbanization and expanding wildlife tourism intensify contact between humans, domestic animals and wildlife, creating complex ecological and behavioural interfaces that facilitate zoonotic (i.e. pathogens originating in animals and spill over to humans) and zooanthroponotic (i.e. pathogens originating in humans and spill over to non-human animals) transmission (Jones et al., 2008). In densely populated urban and touristic settings, practices such as food provisioning and close encounters with free-ranging animals further increase opportunities for cross-species pathogen transmission (Muehlenbein, 2012, Muehlenbein, 2016). These risks are compounded by international tourists’ heightened susceptibility to disease in novel environments (e.g. exposure to novel pathogens, differences in immune status or naïve risk perception) and by their often limited adherence to preventive hygiene measures such as hand hygiene, consumption of safe drinking water and avoidance of animal contact (Muehlenbein and Ancrenaz, 2009; Muehlenbein et al., 2010). Consequently, these settings can drive disease dynamics across ecological and geographical boundaries, spreading pathogens into populations lacking prior exposure or immunity (Muehlenbein and Wallis, 2014; Devaux et al., 2019). Despite the key importance of these rapidly expanding anthropogenic interfaces (i.e. urban and touristic environments), research on pathogen transmission risks across such interconnected human–domestic animal–wildlife systems remains very limited.

Among transmitted pathogens, gastrointestinal (GI) parasites are of particular public health concern (Gallagher et al., 2019). Some protozoan and helminth parasites are associated with chronic illness and immunosuppressive effects in humans (Maizels, 2016), and developmental issues in children (Chifunda and Kelly, 2019), especially in socio-economically disadvantaged settings where health care access is often limited.

In addition to their significant public health impacts, many GI parasites circulate along a host–parasite continuum spanning wildlife, domestic animals and humans (Daszak et al., 2000), raising concerns for animal health and survival within anthropogenically dominated environments. Given their overlapping ecological niches, shared environments and resources, and similar modes of interaction with humans, free-ranging dogs (*Canis lupus familiaris*) and non-human primates (NHPs) are ideal study systems for investigating GI parasite transmission at the human–domestic animal–wildlife interface.

Free-ranging dogs – defined as dogs not under direct human supervision and whose movements are not restricted by human activities, such as feral, stray, community-fed or loosely owned dogs (Cafazzo et al., 2010) – are known vectors and reservoirs for various zoonoses, including GI parasites (Gado et al., 2023, Tomori and Oluwayelu, 2023). In Kathmandu, Nepal, for example, parasite prevalence in free-ranging dogs has been reported at 95–100%, with high levels of polyparasitism including *Toxocara canis, Echinococcus multilocularis* and protozoa such as *Giardia* spp. and *Cryptosporidium* spp. (Adhikari et al., 2023a, 2023b). Their mobility, scavenging behaviour and close association with humans (Majewska et al., 2021) position them as potential environmental amplifiers of parasite contamination, and potential ‘bridge hosts’ in areas also frequented by wildlife, including NHPs. Consequently, they are increasingly monitored as sentinels for both human and wildlife health (Salb et al., 2008; Curi et al., 2016; Bowser and Anderson, 2018).

Like dogs, many NHP species also come into frequent contact with humans, either directly or indirectly through shared environments (Hansen et al., 2023). Tourism-related activities such as provisioning or trekking through NHP habitats can increase cross-species parasite transmission (Borg et al., 2014; Carne et al., 2017; Gillespie and Leendertz, 2006). For example, in Uganda’s Kibale National Park, *Cryptosporidium* spp. and *Giardia* spp. detected in habituated chimpanzees (*Pan troglodytes*) were likely transmitted from tourists due to improper sanitation and close proximity during viewing (Salzer et al., 2007). A recent systematic review reported potential human–macaque parasite transmission in over 25% of studies, involving both direct (e.g. bites, scratches) and indirect (e.g. contaminated surfaces) pathways (Virdana et al., 2024). Beyond tourism, broader anthropogenic pressures also shape parasite transmission risk in NHPs. In urban environments, NHPs often exhibit higher parasite richness than those living in less anthropogenic environments (Thatcher et al., 2018). For instance, studies across Kathmandu reported GI parasite prevalence, including protozoans and helminths, as high as 87–100% in rhesus macaques, *Macaca mulatta* (Tachibana et al., 2013; Sapkota et al., 2020; Tandan et al., 2023; Adhikari et al., 2023b). Furthermore, urban NHPs are often associated with other synanthropic species such as free-ranging dogs, creating opportunities for cross-species parasite transmission through environmental contamination (e.g. faeces) or direct contact (Afonso et al., 2021), although evidence for dog–NHP parasite transmission remains sparse (Cibot et al., 2021).

To date, dyadic transmission risks have been documented between humans and free-ranging dogs (Traub et al., 2002; Dantas-torres and Otranto, 2014; Mulugeta et al., 2019), humans and NHPs (see review in wild Asian NHPs: Patouillat et al., 2024), and dogs and NHPs (Cibot et al., 2021). Yet empirical evidence for triadic human–free-ranging dog–NHP GI parasite transmission remains scarce (Kuthyar et al., 2022), Upadhayay et al., 2026 and the ecological and behavioural mechanisms linking anthropogenic interactions to parasite transmission across this triadic interface are largely unexplored.

GI parasites vary widely in their ecological and biological traits. Protozoa, such as *Giardia* spp., typically have direct life cycles and can replicate rapidly within hosts (Sulaiman and Cama, 2006), whereas helminths, including nematodes and cestodes, often have longer generation times and may require intermediate hosts or environmental development stages (Scott, 2023). Many helminth eggs and protozoan cysts persist for extended periods in soil or water (Scott, 2023). Host specificity ranges from highly specialised taxa to generalist parasites capable of infecting multiple mammalian species (Costello, 2016). Transmission is predominantly faecal–oral (Gallagher et al., 2019), although some helminths rely on trophic or vector-mediated pathways (Scott, 2023). These ecological features shape how parasites may circulate within and between humans, free-ranging dogs and NHPs in shared environments.

Processes such as environmental contamination, intra- and interspecific behaviour, and stress-induced immune modulation are thought to influence parasitic load and infection risk (Kappeler et al., 2015, Beldomenico and Begon, 2016). For instance, in urban vervet monkeys (*Chlorocebus pygerythrus*), individuals engaging more frequently in negative interactions with humans displayed higher anxiety-related behaviours and a greater prevalence of GI parasites (Thatcher et al., 2021). Heightened anxiety can compromise immune function (Düpjan and Dawkins, 2022) and increase susceptibility to parasitic infections (Gillespie et al., 2013), providing an indirect pathway linking anthropogenic disturbance to parasite risk. As urban environments and tourism have both been associated with increased interspecific interactions and related stressors (Maréchal et al., 2011, 2016; Thatcher et al., 2021), it is important to consider how human–dog–wildlife interactions in urban and touristic settings may shape parasite transmission risk.

Understanding cross–species parasite transmission in urban and touristic settings is increasingly important within the One Health approach, yet no previous research has examined behavioural, ecological and cross–species mechanisms simultaneously within a human–dog–NHP triadic interface. The current study addresses this gap by assessing parasite prevalence, diversity, density and overlap among humans, free–ranging dogs and rhesus macaques at Swayambhunath Temple, a major UNESCO World Heritage Site in Kathmandu, Nepal. By treating these three host species as an integrated transmission system rather than isolated dyads, this work offers a novel framework for evaluating zoonotic and zooanthroponotic risks. As such we predict that (1) As free-ranging dogs act as environmental amplifiers of faecal contamination, or ‘bridge hosts’, and exhibit high mobility across human-dominated areas, we predict that dogs will show the highest parasite prevalence and diversity, followed by rhesus macaques, with humans exhibiting the lowest prevalence and diversity; (2) Given that several protozoan and helminth taxa have broad host ranges and environmentally persistent transmission stages, we predict partial overlap in parasite species detected across humans, dogs and rhesus macaques; (3) Because close proximity and intra- and interspecific interactions increase opportunities for exposure to contaminated substrates or infectious stages, we predict that dogs and rhesus macaques that are more frequently recorded near conspecifics or members of another species will exhibit higher parasite diversity and infection intensity; (4) As frequent interactions with humans can elevate anxiety and behavioural stress responses in wildlife (Maréchal et al., 2011; Thatcher et al., 2021), and because stress can impair immune function (Düpjan and Dawkins, 2022), we predict that individuals engaging more frequently in interactions with humans will display higher rates of self-scratching, a behavioural indicator of anxiety (Maestripieri et al., 1992); (5) Finally, as stress induced immune modulation can increase susceptibility to infection, we predict that individuals, i.e. dogs and macaques, exhibiting higher rates of self-scratching will also show higher parasite diversity and infection intensity.

## Materials and methods

### Study site

This study was conducted at Swayambhunath Temple, a UNESCO World Heritage Site in the Northwest of Kathmandu, Nepal (27.7149° N, 85.2904° E) between August 2023 and January 2024. The temple is surrounded by forest, covering a total area of 0.37 km^2^ and is frequented daily by both local and international visitors, who interact with free-ranging dogs (*Canis lupus familiaris*) and rhesus macaques (*Macaca mulatta*) inhabiting the site. Every morning, the study site is swept by the temple management to remove all faeces, dirt and left over food from the main pathways.

### Human participants and animals studied

#### Human participants

A total of 30 participants, who regularly visited the temple, were recruited through opportunistic sampling (19 men, 11 women with an average age of 52 years, ranging from 29 to 75 years). In addition to providing faecal samples for the study, they all completed a questionnaire to assess some demographic information (age, gender, levels of education, employment) as well as the types of interactions they had with dogs and macaques when visiting the temple. All participants provided informed consent prior to their involvement in the study.

#### Dog subjects

Approximately 60 free-ranging dogs live at the site (R. Karki, personal observation). Due to the configuration of the site with non-accessible forest areas and the aggression levels of some dogs, only a subset of dogs was included in the data collection. Therefore, data were collected on 2 groups of free-ranging adult dogs (total of 30 adult dogs): one group located on the temple ground (on top of the hill), and experiencing unpredictable feeding by visitors (20 adult dogs: 6 males and 14 females); the second group located at the entrance of the site (Bhagwan Pau area at the bottom of the hill), and experiencing daily predictable provisioning at 3:30 pm by a local person along with random feeding by visitors throughout the day (10 adult dogs: 5 males and 5 females). Dogs from the 2 groups were not observed to interact with one another.

#### Macaque subjects

It is estimated that over 350 rhesus macaques live at the site aggregated in 4 groups (Ogawa et al., 2024). To match the sample size of the humans and dogs, data were collected from 30 identified adult macaques (16 males and 14 females) from a socially cohesive macaque group. We selected the focal macaques from the largest group at the site, including 219 individuals (87 adult females, 18 adult males) whose home range overlapped with both dog groups and the major walking trail of the visitors. However, most behavioural data on macaques were collected when they were present at the bottom of the hill where visibility was optimal and encounter with aggressive dogs in the surrounded forested areas was reduced. Apart from the predictable provisioning by a local person and unpredictable feeding by visitors (see previous section on dogs), the macaques also rely on natural food resources in the surrounding forested areas.

### Behavioural data collection

Behavioural data were collected on pre-identified adult dogs (by R.K.) and macaques (by S.L.) from August 2023 to January 2024, 3 days per week from 7 a.m. to 7 p.m. Behavioural data were collected using customized data forms created in the HanDBase® application (DDH software) implemented using either Samsung Galaxy F22 or Samsung M01 mobile phones. Observers conducted 10–min focal follows (Altmann, 1974). During each focal session, instantaneous scans were performed at 2–min intervals to record the subject’s behavioural activity (i.e. long-duration behaviours) and to count the number of humans, dogs and conspecifics within 3 m of the focal individual. Therefore, a total of 6 instantaneous scans per 10-min focal session were recorded, and behavioural activities were defined as mutually exclusive. In parallel, short–duration behaviours (i.e. agonistic interactions, approaches, self–scratching and human–animal interactions) were recorded continuously (see ethogram in Table S1, Supplementary Material). Consecutive events of the same behavioural category were considered distinct when separated by approximately 10 sec. Each day focal animals were selected using a predetermined random list.

Data were exported daily into MS Excel. Both observers (R.K. and S.L.) passed inter-observer reliably tests using Cohen’s kappa for each species, and were deemed reliable when Cohen’s kappa was >0.8 (rhesus macaque: Inter-observer reliability value = 0.822; dog: IOR value = 0.865). By the end of the study period, we collected a total of 553 (mean ± s.d. = 19.1 ± 1.6 per individual) focal sessions on dogs and a total of 589 (mean ± s.d. = 19.6 ± 0.76) focal sessions on macaques.

### Faecal sample collection

A total of 180 faecal samples were collected and analysed. Two faecal samples were collected for each individual (humans, dogs and macaques) between August and December 2023 with an average 28 days apart between samples (ranging from 12 to 58 days apart, s.d. = 9.16, s.e. = 0.96). All samples collected (see procedure below) were transported, stored and subsequently analysed at the Central Department of Zoology, Tribhuvan University, Kathmandu.

#### Human samples

Participation in this study was entirely voluntary. Faecal samples were collected from 30 participants using a purposive sampling technique with a non-invasive approach. Prior to sample collection, participants were provided with information about the study and brief instructions on how to collect faecal samples to ensure their informed consent, high-quality sampling and compliance with health and safety procedures. As part of the procedure, participants were provided with gloves, labelled collection vials, cotton, applicator sticks and ziplock bags for sample collection. Participants were instructed to defecate on a clean newspaper placed over the toilet seat. They then collected their faecal sample, avoiding any contaminant such as urine, water or other material, into a tube. The researcher collected faecal samples the following morning and 2.5% potassium dichromate solution was added to preserve the samples.

#### Macaque and dog samples

Fresh faecal samples were collected opportunistically during daily follows in the morning (6–11 a.m.) or evening (3–6 p.m.), immediately after defecation of known individuals. The samples were homogenized, avoiding contamination from any outer elements. Approximately 5 g of faecal material were then stored into a sterile tube containing 2.5% potassium dichromate solution and labelled with the date, time and individual ID.

### Parasitology screening methods

To ensure detection of as wide a range of different parasites as possible, a phase-contrast compound microscopic examination (KERN and Sohn Gmbh OBN 158) was carried out using 3 methods: direct iodine wet mount, sedimentation and flotation methods, including the McMaster methods as described by Zajac et al. (2012). For all methods, except the McMaster, only presence and absence of identified parasites (genus or species level when possible) were recorded.

#### Iodine wet mount

Two drops of faecal sample were placed on a glass slide to which 1 drop of Lugol’s iodine was added and mixed. Then a cover slip was added, and excess fluid was removed using cotton filter paper. The smear was observed under the microscope with a total magnification of 100× and 400×. This technique helps in studying the internal structure and identifying cysts of intestinal protozoa such as *Entamoeb*a spp., *Giardia* spp., *Iodomoeba* spp., *Chilomastix* spp. and *Balantidium* spp. and helminth eggs and larvae (Zajac et al., 2012).

#### Sedimentation method

This method is used to detect eggs of flukes, tapeworms and nematodes whose eggs do not float in common flotation solutions. Firstly, approximately 2 g of the sample was filtered thoroughly via a tea strainer and mixed with 12 mL of water in a 15-mL centrifuge tube. At 1200 rpm, the sample was centrifuged for 5 min. The supernatant was discarded, and the sediment was mixed well. To this sediment, 10 mL of 10% formalin and 3 ml of ethyl acetate was added and again centrifuged at 1200 rpm for 5 min. After the second centrifugation, the supernatant was discarded, leaving only the sediment at the bottom. If sediments were too dry, 1–2 drops of 10% formalin were added and mixed well. A drop of sediment mixed with Lugol’s iodine was placed on a clean slide, covered with a coverslip and observed under microscope at 100× and 400× magnifications (Zajac et al., 2012).

#### Flotation method

This technique is used to float the less dense parasite eggs on a high-density fluid flotation medium. About 2 g of the faecal sample was mixed with 12 mL of water following filtration via a tea strainer in a 15-mL centrifuge tube and then centrifuged at 1200 rpm for 5 min. The supernatant was discarded and the bottom sediment remaining in the tube was mixed well. Then, saturated NaCl solution (6.1 M) was added and filled the tube up to 13 mL and centrifuged again at 1200 rpm for 5 min. After centrifugation, more saturated NaCl was added in the tube until a convex surface was formed at the top, and a clean coverslip was placed over the top of the tube and was left undisturbed for at least 10 min. The coverslip was removed gently, avoiding the dropping of the sample from cover slip and then placed on a clean glass slide. The slide was examined under microscope at a total magnification of 100× and 400× with Lugol’s iodine (Zajac et al., 2012).

#### The McMaster method

The McMaster technique is based on the flotation-based method (see above description), and it is one of the most common and efficient methods for quantifying parasitic elements in faeces, expressed as eggs per gram (EPG) or oocysts per gram (Becker et al., 2016). Approximately 2 g of faecal material were thoroughly mixed with 28 mL of flotation solution to achieve a final volume of 30 mL. The mixture was strained through a tea strainer to remove large debris. Using a dropper, both chambers of the McMaster slide were filled with the filtered solution. The slide was then left undisturbed for at least 5 min to allow sufficient flotation of the parasitic elements. Microscopic examination was performed under 100× and 400× magnification, focusing on the upper layer of the chambers where the eggs and oocysts accumulate. Eggs, oocysts and other parasitic stages observed in each grid lane of both chambers were counted separately to ensure accurate quantification. Only nematodes were observed in our samples using this method; therefore, we calculated the nematode density using the following formula:

EPG = (Total number of eggs counted) × 100 (Total volume (mL)/Examined volume (mL))/2 (g of faeces)

### Data analysis

#### Behavioural data analyses

We used descriptive data to provide some information on demographics and interactions with the different animal species at the site, as well as hygiene measures to put into context the potential parasite transmission risk factors in humans.

Dog and macaque dominance ranks were calculated on the basis of the frequency of decided dyadic aggressive interactions (i.e. those aggressive interactions with a clear winner and loser). As aggressive interactions between dogs were rare (12 events across 553 focal samples; 0.02 events per focal sample) and, in macaques, many aggressive interactions involved group members that could not be reliably identified due to the large group size (296 events across 589 focal samples; 0.5 events per focal sample), we adopted a cautious approach to estimating dominance rank. As such, only the number of aggressive encounters won was used to rank individuals (dogs and macaques) with the other individuals within the group and categorized into high, medium and low ranks according to quartile thresholds: upper quartile (most wins or high rank), interquartile range (moderate number of wins or medium rank) and lower quartile (least number of wins or low rank).

Rates of food provisioning in dogs and macaques were calculated as the total number of events in which a focal animal was fed by people, divided by the total number of focal sessions recorded for that individual.

The average number of humans, dogs and macaques in proximity was calculated as the mean number of individuals recorded within three meters of the focal animal per scan.

Grooming rates (macaques only) were calculated as the number of times a focal animal was recorded engaging in a grooming session, either as groomer or recipient, divided by the total number of focal sessions for that individual.

Rates of human–dog and human–macaque aggression were calculated as the number of events in which the focal animal engaged in an aggressive interaction with a person, either as aggressor or victim, divided by the total number of focal sessions for that individual.

Self-scratching rates were calculated as the total number of self-scratching events recorded from the focal animal divided by the total number of focal sessions recorded on the focal subject.

#### Parasitological data analyses

To ensure the reliability and consistency of the measurement methods, and to avoid the Kappa paradox due to low parasite prevalence, presence/absence repeatability between the two samples was calculated using the PABAK (Prevalence-Adjusted Bias-Adjusted Kappa) (Byrt et al., 1993). Only inter-sampling repeatability was conducted. Eighteen out of twenty parasites had a PABAK outcome over 0.80 (ranging from 0.80 to 0.98), which is considered as almost perfect agreement, only Strongylida (indet.) PABAK was 0.73 and *Strongyloides* spp. PABAK was 0.69, which is considered as substantial agreement (Byrt et al., 1993); therefore, in this study, all the repeated samples were combined for analysis (see Table S2).

To estimate the proportion of parasite species within each sampled host that were detected during our study (i.e. the completeness of our sampling), we carried out rarefaction analysis using the package vegan (Oksanen et al., 2016) in R v 4.4.2 for Mac (R Core Team, 2024) to estimate the proportion of total parasites infecting each species that we are likely to have detected. We used the *specpool* function with default parameters to estimate the extrapolated index of Chao’s diversity for each host species separately. The detection of over 80% of estimated parasite taxa present in each host is considered as comprehensive sampling.

#### Statistical analyses

To test our five predictions, we calculated the following for each individual: (1) infection status which indicates whether parasites were either present (if we recorded the presence of at least one parasite species in at least one of the two samples) or absent; (2) parasite diversity which reflected the number of different parasite taxa detected across both samples; and (3) nematode infection intensity calculated as average EPG between the two samples.

*Prediction 1: parasite prevalence, diversity and nematode infection intensity between the three species*

To compare parasite prevalence between humans, dogs and macaques, we ran a Chi-squared (χ^2^) test of Independence using the *chisq.test* function in R v 4.4.1 (R Core Team, 2024). To assess whether both median parasite diversity and nematode infection intensity differed between the three host species, we ran a Kruskal–Wallis test (due to the violation of normality assumption) using the R function *kruskal.test*.

*Prediction 2: Overlap in parasite species detected in all 3 species*

We used descriptive data and Venn-diagram representation to assess parasite species overlap between humans, dogs and macaques.

*Prediction 3: Intra- and interspecific proximity and interactions association with parasite diversity and nematode infection intensity*

To test whether dog or macaque individuals who were recorded more frequently in proximity or interacting with conspecifics or members of another species are more likely to be infected, or have a higher diversity and infection intensity of parasites, we ran two Generalized Linear Models (GLMs) using the R function *glm*. Parasite diversity and nematode infection intensity were set as dependent variables; we did not run models that included parasite infection status (presence/absence in the faeces) as a dependent variable because the majority of the samples included at least one parasite species (see Results section). Parasite diversity was modelled using a Poisson GLM, while nematode infection intensity was analysed using a Negative Binomial GLM due to overdispersion in the data. Additionally, because EPG values were multipliers of 25, we divided infection intensity values by 25 since GLMs failed to converge when the raw values were included. GLMs included the following predictors (see details in the Behavioural Data Analyses section): rates of provisioning, average number of humans, dogs and macaques within three meters, dominance rank, grooming rates (for macaques only) and rates of human–dog aggression (for dogs only).

To assess collinearity between predictors, we used the *Mypairs* function in the HighstatLibV8 package developed by Zuur (2012). This function carries out pairwise Pearson’s correlation tests between the variables of interest and graphically represents their correlation coefficient *r*. Correlation tests whose *r* > 0.40 were statistically significant and were used to assess which predictors can be included in the models. Following this analysis, for macaques, we did not include human–macaque aggression in the GLM because it was correlated with provisioning rates (Table S3). Furthermore, because, in the macaque dataset, the average number of people in proximity was correlated with both the average number of macaques in proximity and grooming rates, while provisioning rates were correlated with grooming rates (Table S3), for each dependent variable we ran two separate models: one model that included provisioning rates, average number of humans and dogs in proximity and dominance rank; and a second model that included average number of macaques in proximity, grooming rates and dominance rank. We did not find evidence of collinearity in the dog data set (Table S4). None of the models included total rates of human–animal interactions since these were correlated with rates of human–animal aggression and provisioning (Tables S3 and S4).

For all GLMs, we tested the importance of each variable by comparing the full models (i.e. the model with all predictors) with reduced models excluding individual predictors using likelihood ratio tests (LRTs), implemented via the *drop1* function in R.

*Prediction 4: Interactions with humans associated with increased anxiety levels in dogs and macaques*

We tested whether interactions with humans were associated with increased measures of anxiety by running a linear regression that included self-scratching rates as the dependent variable with provisioning rates, average number of people and, for dogs only, human–dog aggression set as predictors. These linear regression models met the assumptions of normality of residuals and homogeneity of variance, which we tested using the *check_model* function within the R ‘performance’ package.

*Prediction 5: Animal’s anxiety levels are related to higher parasite diversity and nematode infection intensity*

Finally, to assess whether animal’s anxiety levels were associated with higher parasite metrics, we ran GLMs that included either parasite diversity (Poisson GLM) or nematode infection intensity (Negative Binomial GLM) as dependent variables, with rates of self-scratching included in all models as predictors.

## Results

### Human demographics and transmission risk factors

All 30 participants regularly visited the temple (24 every day, 2 several times per week, 4 at least once a week), and 20 lived within 1 km radius from the temple. Their education levels ranged from no education (10), primary and secondary levels (19) to bachelor’s degree (1). Their profession varied with 5 civil workers, 13 vendors/business, 4 priests, 7 unemployed or retired and 1 artist. In the survey, 93% of participants reported they always see monkeys when they visit the temple, with 83.3% of participants reporting being within touching distance of the monkeys, and 66.6% of participants reported to have been attacked at least once by the monkeys. Furthermore, 100% participants reported they always see the dogs at the temple, 80% of the participants reported to be within touching distance of dogs and 36.6% reported to have been attacked at least once by the dogs at the temple. None of the participants reported to wear a mask or gloves when interacting with the monkeys or the dogs, and 76.6% said they would visit the temple when they are feeling unwell if they were able to. Four participants reported they had taken medicine for intestinal helminths in the last 6 months.

### Parasite sample repeatability and rarefaction

Overall, sample repeatability was very high (0.83–0.98) in 90% of the parasites detected, and moderately high (0.63–0.73) in 10% of the parasites detected (see Table S2). Therefore, the repeated samples were combined for analysis, and no parasite taxon was excluded from analysis.

**Figure 1.**
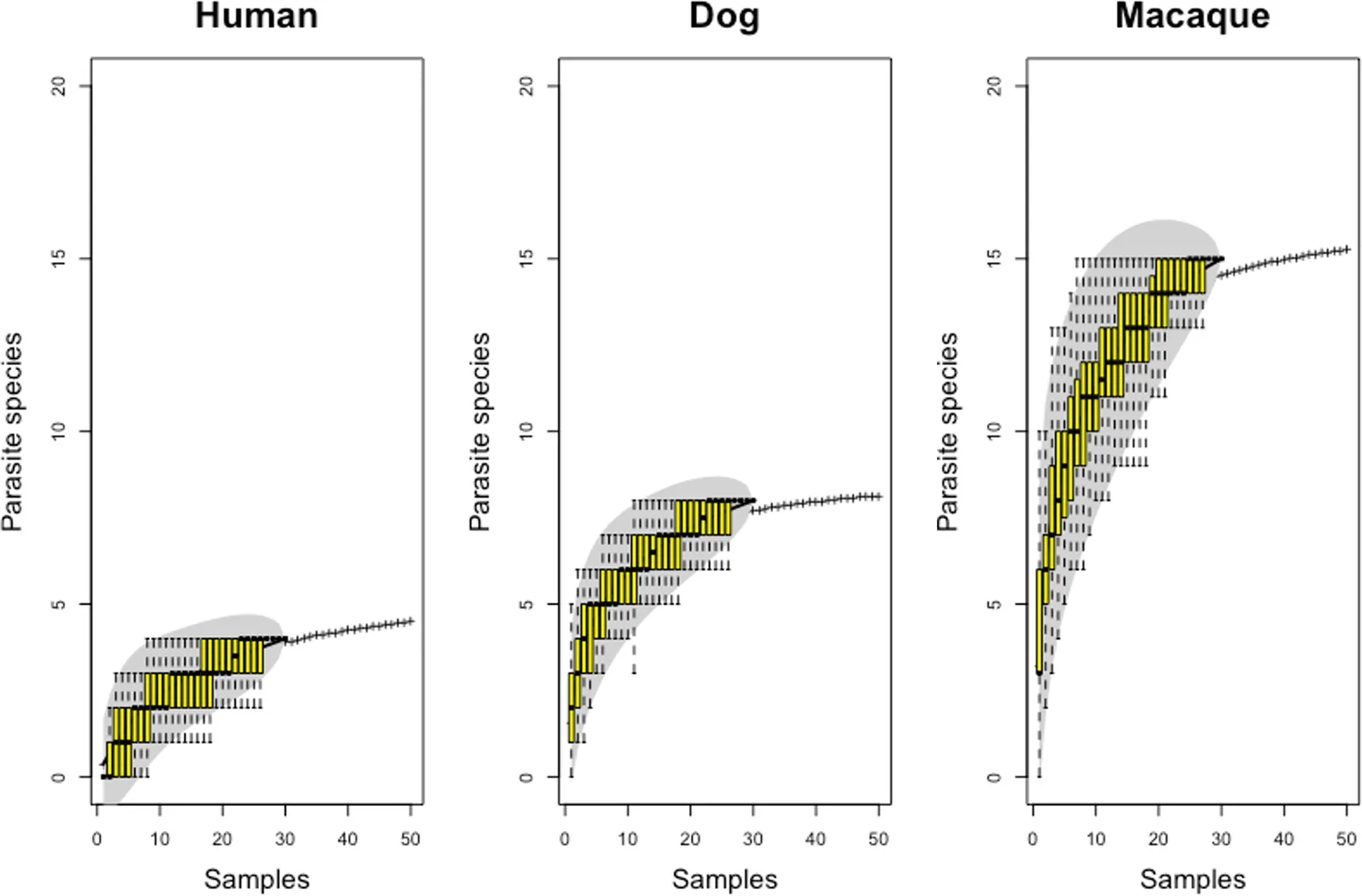
Predicted species accumulation curves for each host species based on the accumulation of parasite species. Predicted points, denoted by ‘+’, are overlaid by confidence intervals (grey shading) and barplots from raw data based on 100 permutations of adding data in a random order.

Rarefaction analysis predicted that we detected over 80% of parasite taxa present in each host (Table 1; Figure 1); therefore, the sampling was considered as comprehensive.

**Table 1.** Extrapolated Chao’s index of parasite richness (mean ± 1 s.e.) for each host species, along with observed parasite richness (number of parasite categories recorded) Table 1 long description.

Species	Extrapolated richness	Observed richness
Macaque	17.175 ± 3.294	15
Human	4.967 ± 2.102	4
Dog	9.933 ± 3.626	8

### Prediction 1: Parasite prevalence, diversity and overlap in humans, dogs and rhesus macaques

We found a significant difference in parasite prevalence (Chi-square test of Independence: χ^2^_2_ = 43.6, *P* < 0.001), diversity (Kruskal–Wallis test: χ^2^_2_ = 54.8, *P* < 0.001) and nematode infection intensity (Kruskal–Wallis test: χ^2^_2_ = 30.9, *P* < 0.001) across the 3 species. More specifically, we detected at least 1 parasite in the majority of faecal samples of dogs (27/30 or 90%) and macaques (29/30 or 97%), whereas we found parasites in only 28% (8/30) of human samples (Table 2). Contrary to our prediction, macaques displayed a significantly higher diversity of parasites than both dogs (Mann–Whitney *U* test: *U* = 169, *P* < 0.001) and humans (*U* = 30.5, *P* < 0.001), with dogs showing higher parasite diversity than humans (*U* = 793.7, *P* < 0.001, Figure S1). Finally, human samples showed a significantly lower nematode infection intensity than samples collected from both dogs (*U* = 685, *P* < 0.001) and macaques (*U* = 106, *P* < 0.001), although there was no difference in nematode infection intensity between macaques and dogs (*U* = 340.5, *P* = 0.101, Figure S2).

**Table 2.** Occurrence (number of samples), prevalence (%), diversity and nematode infection intensity of endoparasites recorded in human, dog and macaque samples Table 2 long description.

Groups	GIPs	Morphological form	Human	Dog	Macaque
Protozoan	*Balantioides/Buxtonella*-like ciliate	Cyst and trophozoite	0	0	21
	*Blastocystis* spp.	Cyst	0	0	1
	*Chilomastix mesnili*	Cyst	0	0	3
	*Cystoisospora* spp. (*Isospora* spp.)	Oocyst	0	0	1
	*Endolimax* spp.	Cyst	0	0	2
	*Entamoeba coli*	Cyst	0	0	9
	*Entamoeba* spp.	Cyst	4	2	31
	*Giardia lamblia*	Cyst	1	0	0
	*Iodamoeba buetschlii*	Cyst	1	0	4
Cestodes	*Bertiella* spp.	Egg	0	0	1
	*Dipylidium caninum*	Egg	0	5	0
Nematodes	*Ascaris* spp.	Egg	5	1	0
	*Enterobius* spp.	Egg	0	0	2
	*Physaloptera* spp.	Egg	0	0	3
	Strongylida (indet.)	Egg	0	18	25
	*Strongyloides* spp.	Egg and larvae	0	21	20
	*Toxascaris leonina*	Egg	0	1	0
	*Toxocara canis*	Egg	0	7	0
	*Trichuris* spp.	Egg	0	8	6
Prevalence: Percentage (%) of individuals with at least 1 parasite			26.67	90	96.67
Average parasite diversity (standard error)			0.37 (0.12)	2.03 (0.25)	4.30 (0.50)
Average nematode infection intensity eggs per 1/25th of a gram (McMaster methods only) (standard error)			5.83 (3.73)	61.67 (13.55)	89.17 (14.32)

GIPs, gastrointestinal parasites.

### Prediction 2: Parasite overlap in humans, dogs and rhesus macaques

**Figure 2.**
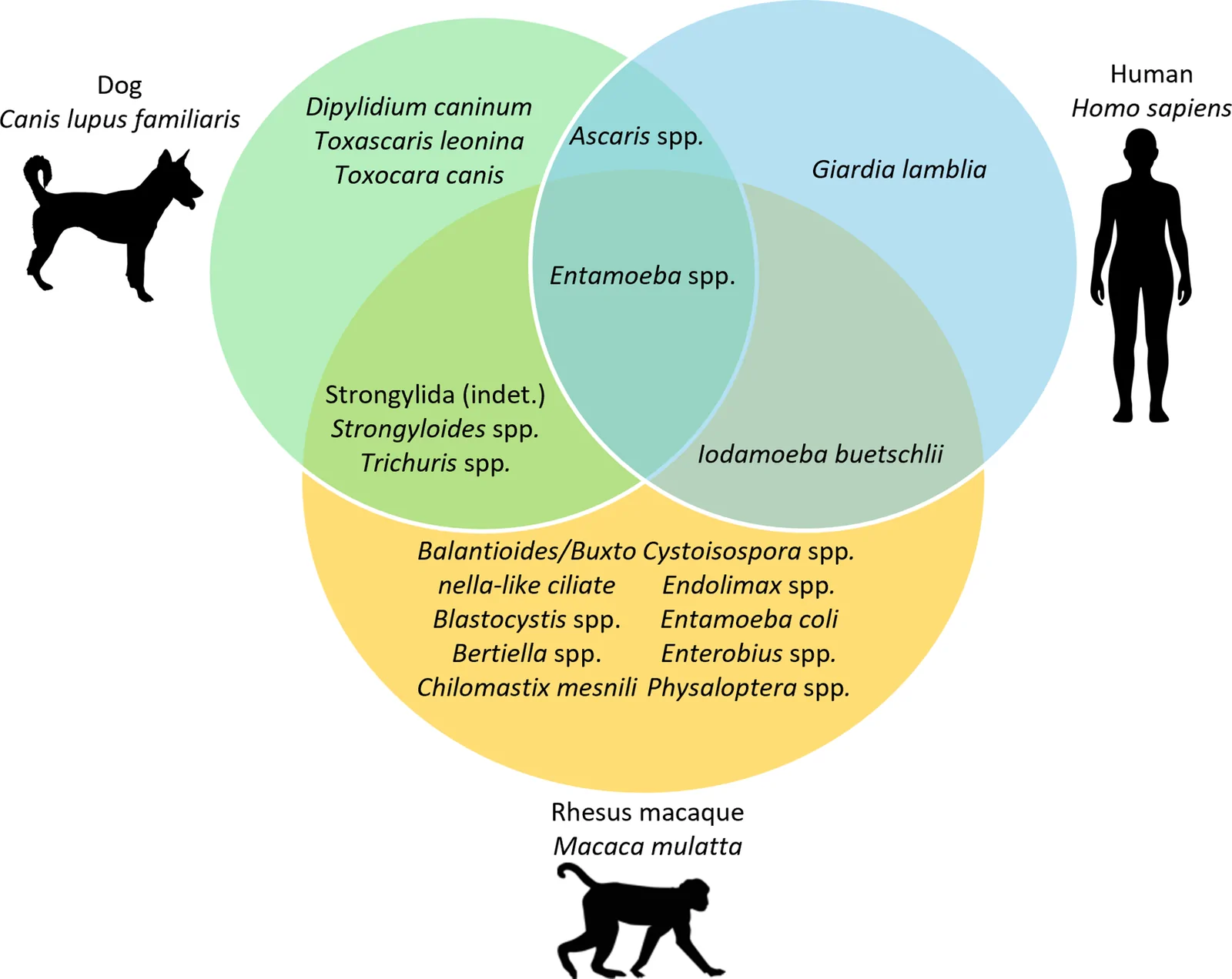
Venn-diagram showing parasite prevalence and overlap for each species. Figure 2 long description.

All three species had one parasite in common (*Entamoeba* spp.), but some other parasites were found in two species: *Ascaris* spp. in humans and dogs; *Iodamoeba buetschlii* in humans and macaques; and Strongylida (indet.), *Strongyloides* spp. and *Trichuris* spp. in dogs and macaques (Figures 2 and S3).

### Prediction 3: Drivers of parasite diversity and nematode infection intensity in animals

Our GLM analysis indicated no effect of provisioning rates, dominance rank, human–dog aggression or proximity to humans, macaques or other dogs on either parasite diversity or nematode infection intensity in dogs (Table 3).

**Table 3.** Results of likelihood ratio tests (LRTs) assessing the contribution of each predictor to the full GLMs Table 3 long description.

Outcome (Model)	Predictors	d.f.	Deviance	AIC	LRT	*P*-value
Parasite diversity (Poisson GLM)	Null model		20.3	105.7		
	Rates of provisioning	1	21.0	104.5	0.7	0.392
	Average number of macaques in proximity	1	20.5	103.9	0.1	0.704
	Average number of humans in proximity	1	21.7	105.1	1.4	0.243
	Average number of dogs in proximity	1	20.3	103.8	0.0	0.894
	Rates of human–dog aggression	1	20.5	103.9	0.2	0.665
	Rank	2	20.5	102.0	0.2	0.903
Nematode infection intensity (Negative Binomial GLM)	Null model		31.9	135.1		
	Rates of provisioning	1	32.1	133.3	0.14	0.710
	Average number of macaques in proximity	1	31.9	133.2	0.02	0.898
	Average number of humans in proximity	1	33.2	134.4	1.23	0.267
	Average number of dogs in proximity	1	32.0	133.2	0.03	0.855
	Rates of human–dog aggression	1	32.3	133.6	0.42	0.517
	Rank	2	32.3	131.6	0.45	0.800

AIC, Akaike Information Criterion.

Each row shows the effect of removing 1 predictor from the full model.

**Figure 3.**
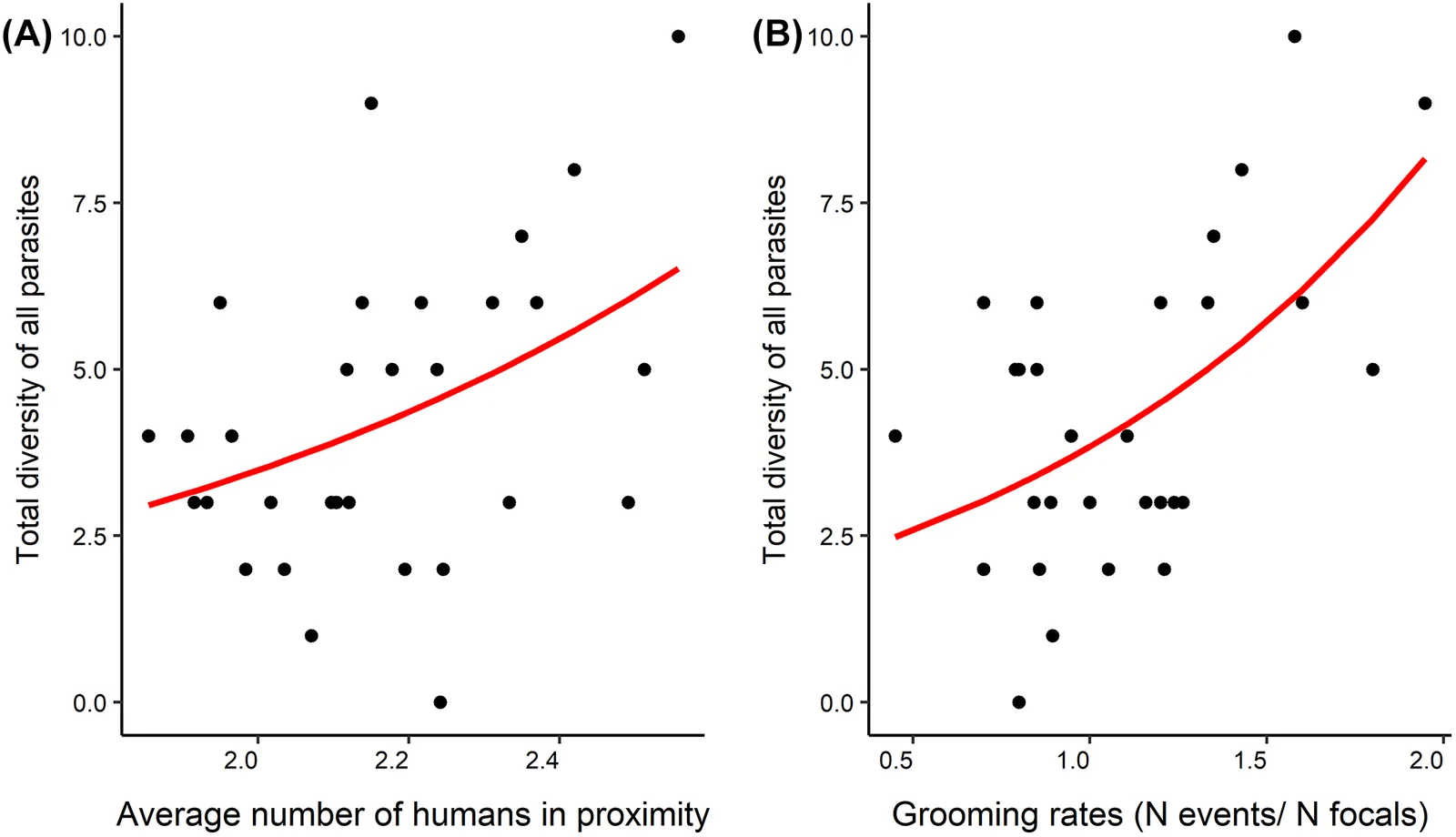
Scatterplots plotting the total diversity of parasites in macaques’ samples against (A) average number of humans in proximity of the macaques and (B) macaque grooming rates. Red lines represent the predicted values (from the GLMs).

Our Poisson GLM analysis revealed a significant effect of average number of humans, grooming rates and dominance rank on parasite diversity in macaques (Table 4). This analysis indicated that macaques who were more frequently in proximity with, on average, a larger number of people and groomed each other more frequently displayed higher parasite diversity (Figure 3).

**Table 4.** Results of likelihood ratio tests (LRTs) assessing the contribution of each predictor to the full GLMs Table 4 long description.

Outcome (Model)	Predictors	d.f.	Deviance	AIC	LRT	*P*-value
Parasite diversity (Poisson GLM)	Null model		22.9	129.2		
	Rates of provisioning	1	22.9	127.2	0.0	0.966
	**Average number of humans in proximity**	**1**	**27.3**	**131.6**	**4.4**	**0.036**
	Average number of dogs in proximity	1	25.5	129.8	2.6	0.109
	**Rank**	**2**	**32.3**	**134.7**	**9.5**	**0.009**
Parasite diversity (Poisson GLM)	Intercept		24.9	129.2		
	**Rates of grooming interactions**	**1**	**31.1**	**133.4**	**6.2**	**0.013**
	Average number of macaques in proximity	1	25.0	127.3	0.1	0.755
	Rank	2	28.3	128.6	3.4	0.182
Nematode infection intensity (Negative Binomial GLM)	Null model		35.8	146.0		
	Rates of provisioning	1	37.2	145.4	1.39	0.238
	**Average number of humans in proximity**	**1**	**44.2**	**152.4**	**8.33**	**0.004**
	Average number of dogs in proximity	1	35.9	144.1	0.04	0.835
	Rank	2	38.0	144.2	2.18	0.336
Nematode infection intensity (Negative Binomial GLM)	Intercept		34.9	148.8		
	Average number of macaques in proximity	1	37.4	149.3	2.51	0.113
	Rates of grooming interactions	1	34.9	146.8	0.002	0.968
	Rank	2	37.4	147.3	2.49	0.288

AIC, Akaike Information Criterion.

Each row shows the effect of removing 1 predictor from the full model. Significant predictors are indicated in bold.

**Figure 4.**
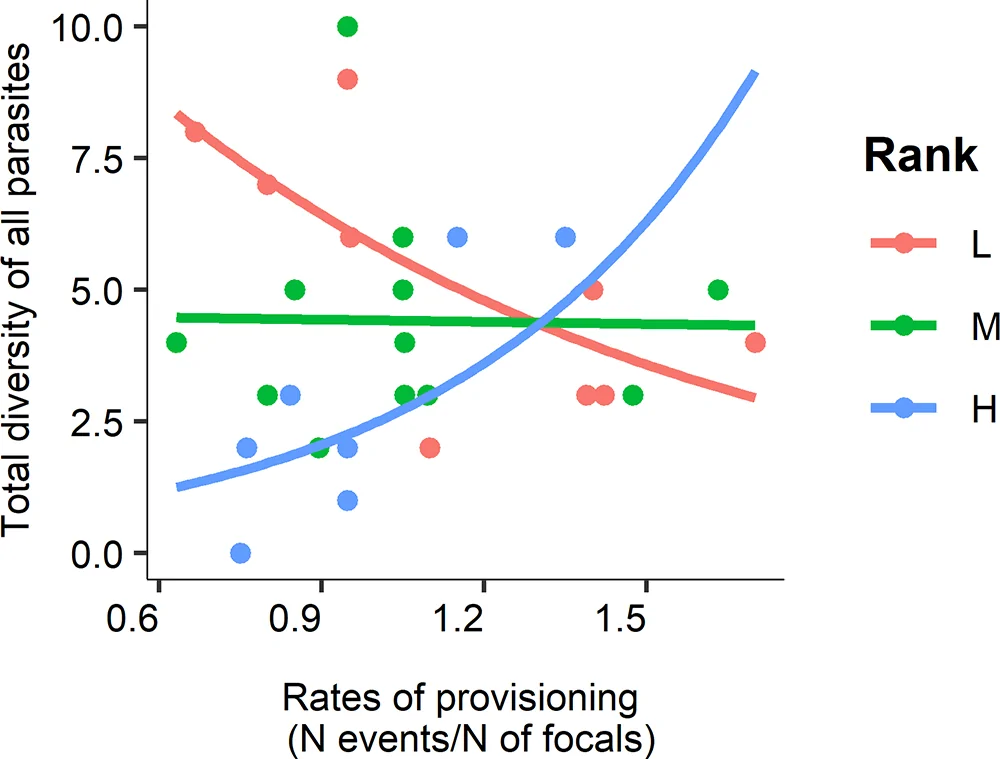
Scatterplot plotting the total diversity of parasites in macaques’ samples against the interaction between provisioning rates and rank. Lines represent the predicted values (from the Poisson GLM) of total parasite diversity in relation to variation in rates of provisioning for high- (blue), middle- (green) and low- (red) ranking macaques.

As we found that dominance rank had a significant effect only in one of the two models, we tested whether dominance rank might have influenced parasite diversity by interacting with either provisioning rates or proximity to humans or dogs. This analysis revealed that there was a significant interaction between dominance rank and provisioning rates (LRT = 9.0, *P* = 0.011). More specifically, while high-ranking macaques who were more frequently provisioned by people displayed higher parasite diversity, low-ranking macaques who were *less* frequently provisioned by people displayed higher parasite diversity (Figure 4).

**Figure 5.**
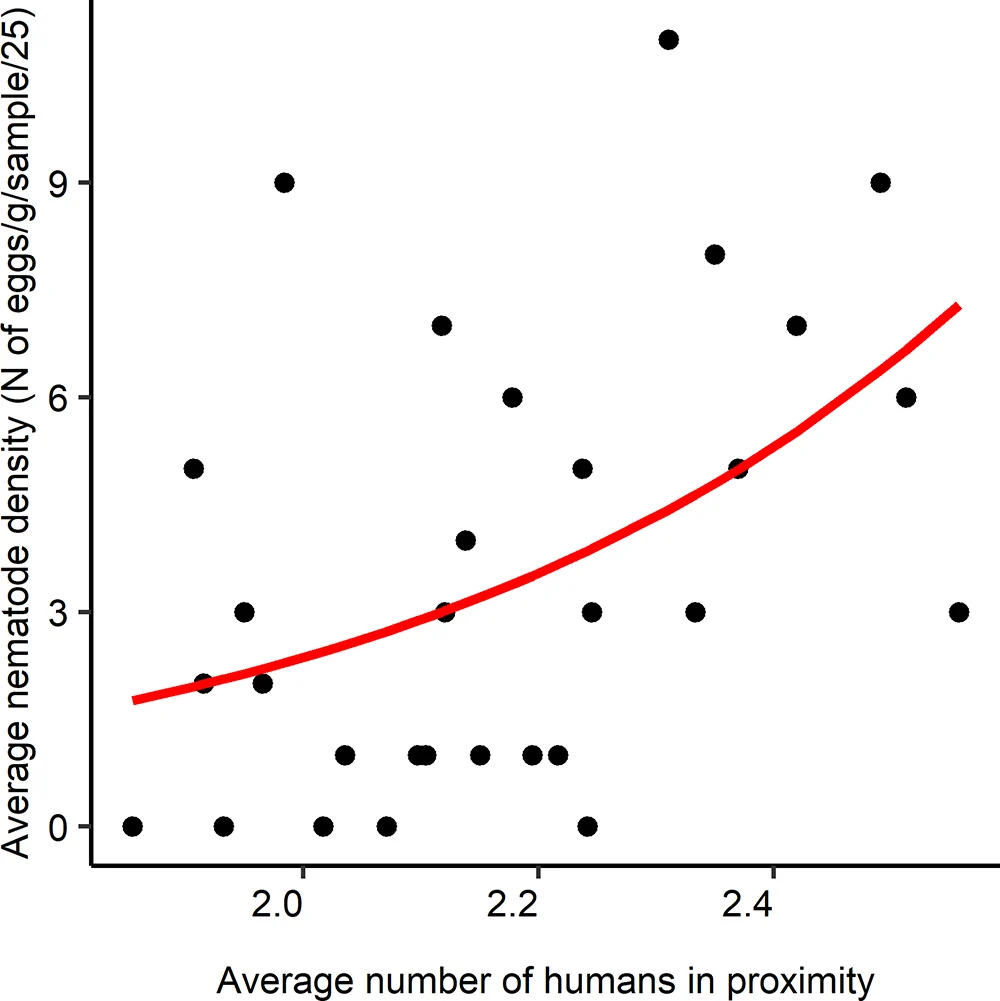
Scatterplot plotting average nematode infection intensity (between the two samples) in macaques’ samples against average number of humans in proximity of the macaques. Red line represents the predicted values (from the Negative Binomial GLM) of total parasite diversity in relation to variation in average number of humans in proximity.

When we tested the effect of the predictors on nematode infection intensity, we, again, found a significant positive effect of average number of people in proximity (Table 4 and Figure 5). However, none of the other variables were significantly associated with nematode infection intensity (Table 3).

### Prediction 4: Self-scratching rates and human–animal interactions

**Figure 6.**
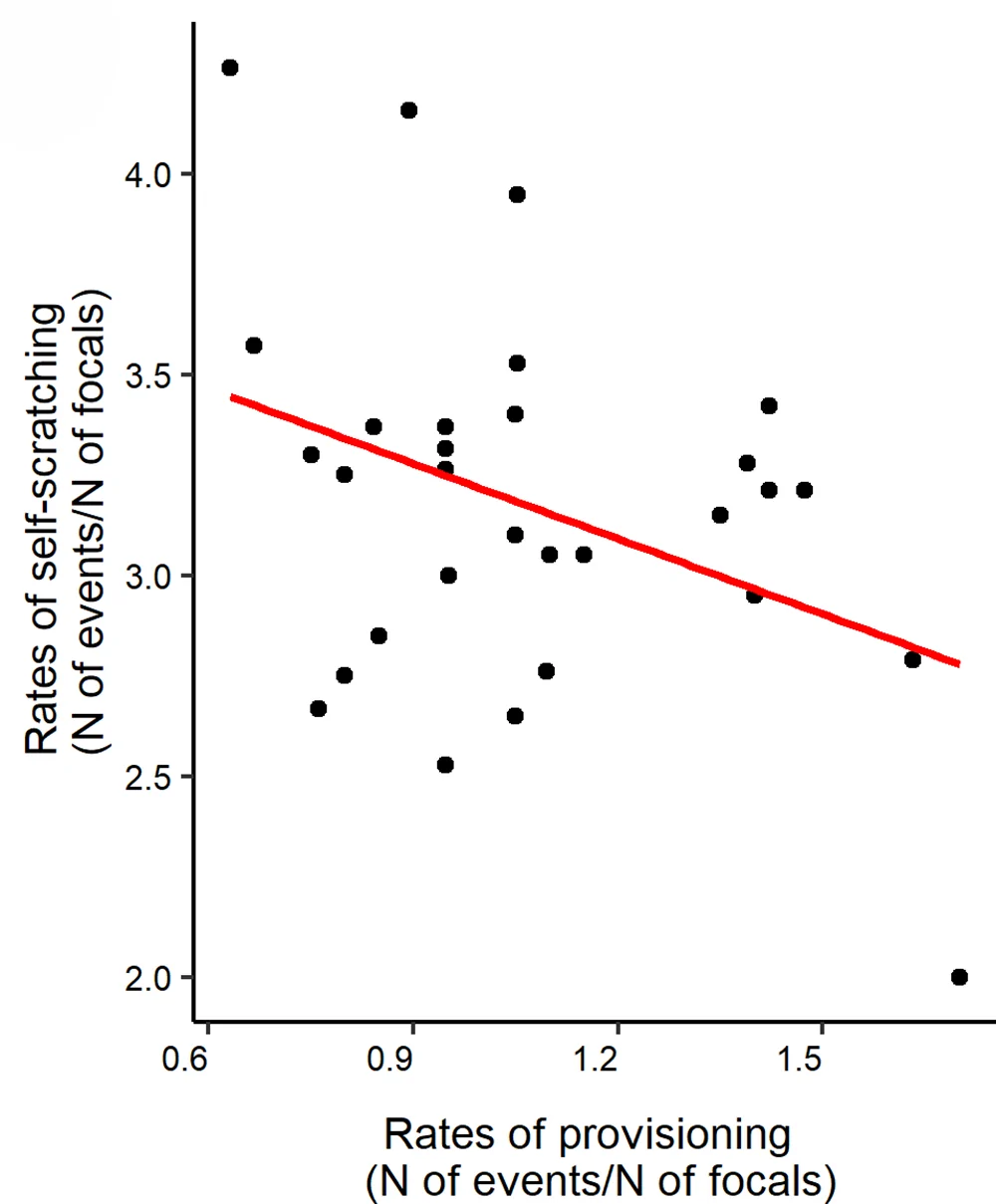
Scatterplot plotting rates of self-scratching against rates of provisioning in macaques. Red line represents the best fit line.

Our regression model showed no significant effect of human–dog interaction, proximity to humans or rank on dogs’ self-scratching rates (Table 5). However, we found a negative association between rates of provisioning and rates of self-scratching in macaques (Table 5), indicating that macaques who received food less frequently from people self-scratched more frequently (Figure 6).

**Table 5.** Results of likelihood ratio tests (LRTs) assessing the contribution of each predictor to the full linear regression model testing the effect of: Dogs (rates of provisioning, average number of humans in proximity, rates of human–dog aggression and rank on rates of self-scratching); macaques (rates of provisioning, average number of humans in proximity and rank on rates of self-scratching) Table 5 long description.

Outcome (Model)	Predictors	d.f.	Sum of square	RSS	AIC	*P*-value
	Null model			1.12	−82	
Dog	Rates of provisioning	1	0.02	1.14	−84	0.437
RSS	Average number of humans	1	0.00	1.12	−84	0.952
(Linear regression)	in proximity					
	Rates of	1	0.02	1.14	−84	0.460
	human–dog aggression					
	Rank	2	0.06	1.18	−85	0.470
Macaque	Null model			4.44	−47	
Rates of self-scratching	**Rates of provisioning**	**1**	**0.91**	**5.35**	**−44**	**0.018**
(Linear regression)	Average number of humans	1	0.62	5.06	−47	0.141
	in proximity					
	Rank	2	0.36	4.80	−47	0.125

AIC, Akaike Information Criterion; RSS, rates of self-scratching.

Each row shows the effect of removing 1 predictor from the full model.

### Prediction 5: Self-scratching rates and parasite diversity and nematode infection intensity in animals

**Figure 7.**
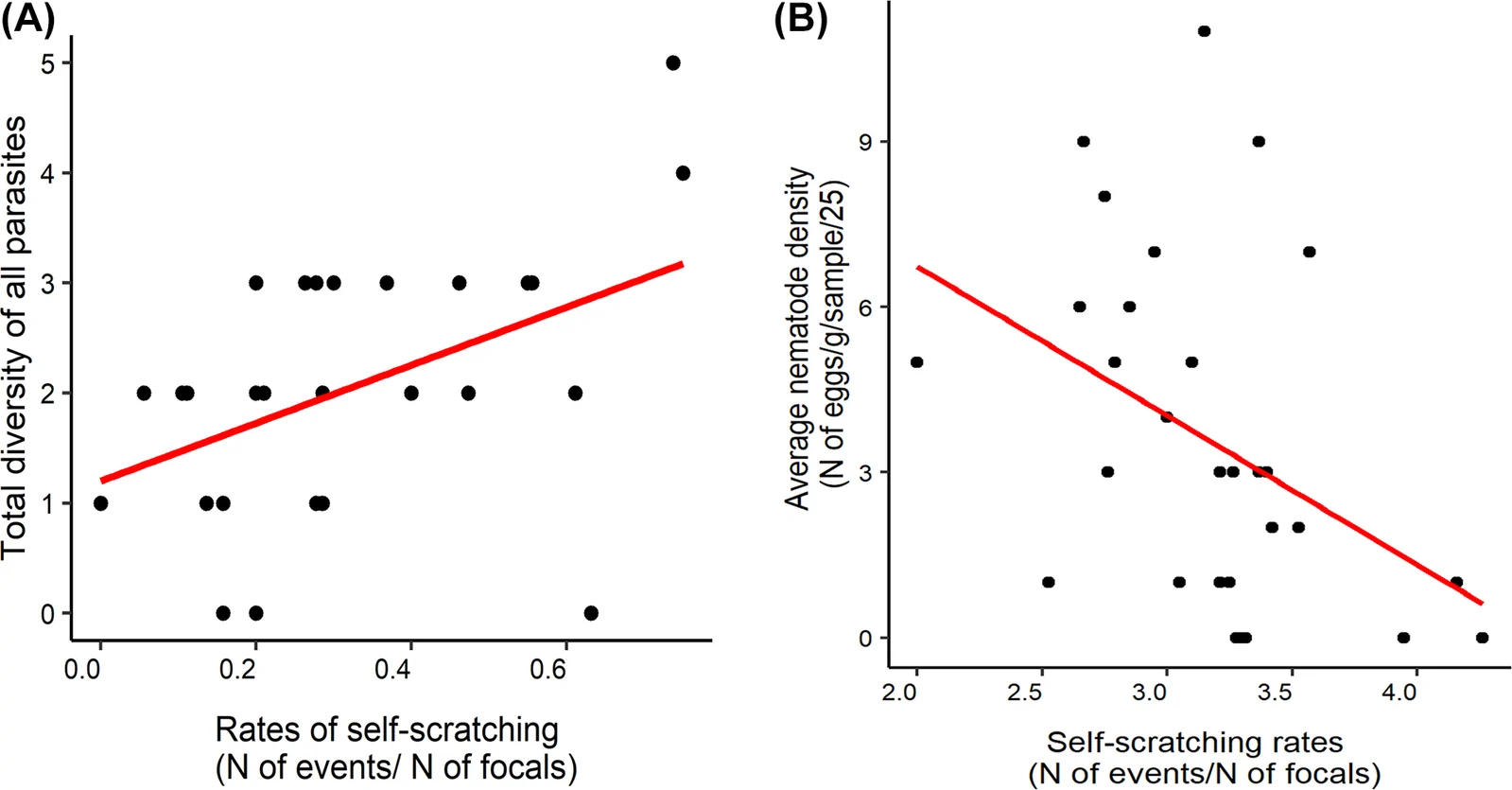
Scatterplots plotting: (A) total diversity of all parasites against rates of self-scratching in dogs and (B) average nematode infection intensity (between the two samples) in macaques’ samples against rates of self-scratching in macaques. Red lines represent the best fit line.

Our regression model showed a positive relationship between self-scratching rates and parasite diversity in dogs (Estimate: 1.21, s.e. = 0.60, *Z* = 2.0, *P* = 0.045, Figure 7A), whereas self-scratching rates did not predict nematode infection intensity (Estimate: 1.21, s.e. = 1.18, *Z* = 1.02, *P* = 0.308).

In macaques*, w*hile self-scratching rates did not predict parasite diversity (Estimate: −0.19, s.e. = 0.20, *Z* = −0.96, *P* = 0.336), we did find a negative relationship between self-scratching rates and nematode infection intensity (Estimate: −0.96, s.e. = 0.36, *Z* = −2.65, *P* = 0.008; Figure 7B).

## Discussion

Our study provides the first integrated assessment of parasite prevalence, diversity, density and overlap across human–dog–macaque systems in an urban and highly touristic environment. Overall, parasite metrics differed across species (i.e. GI parasite prevalence, diversity, density and overlap), with, for example, macaques harbouring the highest parasite prevalence and diversity, followed by dogs and then humans, highlighting potential asymmetries in cross-species transmission. While humans had the lowest parasite prevalence, diversity, nematode density and overlap, they presented some behavioural practices that have the potential to increase exposure risk. In addition, intra- and interspecies interactions as well as stress-related behaviours showed species-specific relationships with parasite diversity and nematode infection intensity, highlighting the importance of exploring behavioural, ecological and cross–species mechanisms simultaneously. These results emphasize that urban environments and tourism not only facilitate direct pathogen transmission between human and animal populations but can also indirectly affect parasite risk through behavioural and social mechanisms.

Our findings revealed key behavioural risk factors that may contribute to parasite transmission to humans. Human participants reported frequent proximity and direct contact with macaques and dogs, combined with low use of protective measures (e.g. masks or gloves), all behaviours associated with increase pathogen transmission risk (Penakalapati et al., 2017). The high rates of reported attacks, particularly by macaques, further underscore the risk of injury and pathogen transmission (Carne et al., 2017; Virdana et al., 2024). Despite this, few participants had recently taken antiparasitic medication, and most indicated they would still visit the temple when unwell. These behaviours mirror patterns at other tourist sites worldwide (Engel et al., 2006; Muehlenbein and Ancrenaz, 2009; Muehlenbein et al., 2010) and highlight the need for targeted public health interventions and messaging to promote safe human–animal interactions in culturally significant urban spaces.

Contrary to the first prediction, macaques, but not dogs, exhibited higher parasite diversity compared to dogs and humans. However, nearly all macaque and dog samples tested positive for at least one parasite, supporting their potential role as reservoir hosts for human infection and highlighting their value as important sentinel species (Salb et al., 2008; Patouillat et al., 2025). Despite differences in parasite diversity, nematode density did not differ significantly between dogs and macaques, suggesting shared environmental transmission pathways in these species as found in other species-specific studies on macaque species (MacIntosh, 2014; Patouillat et al., 2024) and free-ranging dogs (Camacho-giles et al., 2024). Lower parasite metrics in humans likely reflect better hygiene and reduced environmental contact, which would limit faecal–oral transmission compared to free-ranging animals (Penakalapati et al., 2017).

Parasite overlap supported the second prediction, particularly for *Entamoeba* spp., detected across all three species. This finding aligns with direct faecal–oral or environmentally mediated transmission. Although molecular confirmation was not possible, previous research has provided evidence of cross-species transmission of *Entamoeba* spp. between NHPs and humans (Levecke et al., 2015; Deere et al., 2019) and dogs (Dini et al., 2025). Additional overlaps occurred between dyadic hosts including humans and dogs (*Ascaris* spp.), humans and macaques (*Iodamoeba buetschlii*) and dogs and macaques (Strongylida (indet.), *Strongyloides* spp., *Trichuris* spp.). However, parasites requiring intermediate hosts (e.g. *Dipylidium caninum, Bertiella* spp., *Physaloptera* spp.) did not overlap. These findings reinforce the evidence for direct faecal–oral or environmentally mediated transmission pathways, highlighting the zoonotic and zooanthroponotic risks associated with close human–animal contact and shared spaces in densely populated urban and touristic environments.

The results indicated species-specific evidence in support of the link between social and anthropogenic drivers of parasite diversity and nematode infection intensity. While in dogs, these parasite metrics were not shaped by any of these factors, in macaques, individuals in proximity to larger numbers of humans and those engaging in more frequent grooming exhibited higher parasite diversity. In contrast, nematode infection intensity was primarily associated with the average number of people in proximity, with no effect of grooming, dominance rank or provisioning.

In dogs, these findings suggest that high environmental contamination may overshadow behavioural effects, consistent with findings in other free-ranging dog populations (Camacho-giles et al., 2024). However, in macaques, these results might reflect the simultaneous role of both human–animal interactions (Thatcher et al., 2021) and social contact (MacIntosh et al., 2012) in shaping parasite transmission risk. Specifically, the significant interaction between dominance rank and provisioning rates suggests a complex relationship: while high-ranking macaques that were frequently provisioned by people had increased parasite diversity, low-ranking individuals with lower provisioning rates also exhibited high parasite diversity, potentially reflecting a complex interplay between alternative transmission pathways, social and environmental stress (Kappeler et al., 2015). This highlights the importance of anthropogenic influences, particularly human presence and food provisioning, in shaping parasite burdens in urban wildlife populations, while also illustrating how social dynamics may mediate these effects. Together, the outcomes underscore differences in behavioural and environmental risk factors between overlapping animal species.

Similarly to the third prediction above, species-specific evidence supported the association between social or anthropogenic factors and self-scratching behaviour, a common indicator of anxiety. Again, none of these factors were associated with self–scratching rates in dogs. However, individual macaques that received less provisioning from humans scratched more frequently. Mixed evidence has been found in other NHPs, with increased self-scratching rates associated with food provisioning interactions in Barbary macaques (Maréchal et al., 2016) and no association between these variables in vervet monkeys (Thatcher et al., 2021). This mixed evidence might highlight the interplay of environmental, social and individual factors on the occurrence of self-scratching behaviour (Duboscq et al., 2016).

Species-specific differences were also found when exploring the relationships between self-scratching and GI parasite diversity and nematode infection intensity. In dogs, self-scratching rates were positively associated with parasite diversity but showed no relationship with nematode infection intensity. In macaques, by contrast, self-scratching was negatively associated with nematode infection intensity, but it was not related to parasite diversity. Therefore, similarly to dogs, this suggests that self-scratching behaviours may interact with host immunity or transmission pathways in complex ways (Cope et al., 2019). The synergy between nutritional status, especially dietary stress and parasite loads is well known (Chapman et al., 2006). However, while dietary stress is typical lower in urban environments with greater food availability, Thatcher et al. (2021) still found increases in both anxiety–related behaviour and parasite metrics, which aligns with the dogs but not the macaques’ outcomes. This suggests that other individual, social or environmental stressors in urban and touristic settings may shape the relationship with parasite diversity and nematode infection intensity differently in the two species. These stressors might provide some negative consequences for the long-term health and survival of both dogs and macaques. Although rhesus macaques are currently listed as Least Concern (Singh et al., 2024), their fragmented distribution around major touristic temples in Kathmandu makes their health and long–term viability a concern.

In conclusion, our study shows that pathogen transmission risk in urban and touristic environments is shaped by complex interactions between humans, dogs and macaques. Both animal species may act as reservoirs and sentinels of GI parasite exposure, underscoring their relevance for public health surveillance. Importantly, anthropogenic influences, particularly tourism and food provisioning, can indirectly shape parasite risk by altering host behaviours like anxiety-related behaviours in macaques. In addition, such behaviours were associated with some parasite metrics in both dogs and macaques. These might have negative impacts on the health and survival of animal species living alongside humans. Integrating behavioural ecology and human-mediated interactions into urban disease ecology frameworks is therefore essential for understanding and mitigating pathogen transmission in densely shared human–domestic animal–wildlife landscapes.

## Supporting information

10.1017/S0031182026102339.sm001 K C et al. supplementary material 1 K C et al. supplementary material

10.1017/S0031182026102339.sm002 K C et al. supplementary material 2 K C et al. supplementary material

## Data Availability

The anonymised data are publicly available through the Lincoln Repository https://doi.org/10.24385/lincoln.32568081.

## Supplementary material

The supplementary material for this article can be found at https://doi.org/10.1017/S0031182026102339.

## Statement of inclusion

Our study brings together authors from a number of different countries, including scientists based in the country where the study was carried out. All authors were engaged early on with the research and study design to ensure that the diverse sets of perspectives they represent was considered from the onset. Whenever relevant, literature published by scientists from the region was cited; efforts were made to consider relevant work published in the local language.

## Figure 2 Long description

The Venn diagram illustrates the overlap of parasites among three species: dogs, humans and rhesus macaques. The left circle represents dogs, labeled with ′Canis lupus familiaris′ and includes parasites such as Dipylidium caninum, Toxascaris leonina and Toxocara canis. The right circle represents humans, labeled with ′Homo sapiens′ and includes Giardia lamblia. The bottom circle represents rhesus macaques, labeled with ′Macaca mulatta′ and includes various parasites like Balantioides, Cystoisospora spp. and Endolimax spp. The overlapping regions show shared parasites: Ascaris spp. is common to dogs and humans; Entamoeba spp. is shared by all three species; Iodamoeba buetschlii is found in humans and macaques; Strongylida (indet.), Strongyloides spp. and Trichuris spp. are shared by dogs and macaques. The diagram effectively highlights the specific and shared parasitic infections among the three species.

Navigate back to Figure 2.

## Table 2 Long description

The table lists gastrointestinal endoparasites by group and species, their observed morphological form, and the number of positive samples in humans, dogs, and macaques. Overall infection prevalence was 26.67 percent in humans, 90 percent in dogs, and 96.67 percent in macaques. Average parasite diversity was lowest in humans at 0.37, higher in dogs at 2.03, and highest in macaques at 4.30. Nematode infection intensity measured as eggs per one twenty-fifth of a gram was lowest in humans at 5.83, higher in dogs at 61.67, and highest in macaques at 89.17. Macaques showed many protozoa, including Balantioides or Buxtonella-like ciliates with 21 positives and Entamoeba species with 31, plus several other protozoa at lower counts. Dogs were dominated by nematodes, especially Strongyloides with 21 positives and Strongylida with 18, and also had cestode Dipylidium caninum with 5 positives. Humans had fewer detections overall, mainly Ascaris with 5 positives and Entamoeba species with 4, with single detections of Giardia lamblia and Iodamoeba buetschlii. Counts reflect detected positives in the sampled groups and do not indicate severity beyond the separate intensity measure, which is reported only for nematodes using the McMaster method.

Navigate back to Table 2.

## Table 1 Long description

The table reports parasite richness for three host species using an extrapolated estimate with a one standard error range, alongside the number of parasite categories actually recorded. Macaques have the highest estimated richness at about 17.2 with an uncertainty of about 3.3, and 15 categories observed. Dogs are intermediate, with an estimated richness near 9.9 with an uncertainty of about 3.6, and 8 observed. Humans have the lowest values, with an estimated richness near 5.0 with an uncertainty of about 2.1, and 4 observed. Across all species, the estimated richness is slightly higher than the observed count, suggesting additional parasite categories may be present but not detected. The uncertainty ranges are relatively large, especially for dogs and macaques, so differences should be interpreted cautiously.

Navigate back to Table 1.

## Table 3 Long description

The table reports likelihood ratio tests from two generalized linear models, one for parasite diversity using a Poisson model and one for nematode infection intensity using a negative binomial model. For each outcome, rows show the change in deviance and AIC when a single predictor is removed from the full model, along with the test statistic and p-value. In the parasite diversity model, removing any predictor yields small test statistics from 0.0 to 1.4 and p-values from 0.243 to 0.903, indicating no clear contribution from provisioning rate, proximity to macaques, humans, or dogs, human-dog aggression, or rank. The largest signal in this model is for average number of humans in proximity, but its p-value remains 0.243. In the nematode intensity model, test statistics are also small, from 0.02 to 1.23, with p-values from 0.267 to 0.898, again suggesting no predictor meaningfully improves model fit. The largest signal here is also average number of humans in proximity, with p-value 0.267. Null-model deviance and AIC are 20.3 and 105.7 for parasite diversity and 31.9 and 135.1 for nematode intensity; changes across predictor removals are modest. These results indicate limited evidence for effects of the listed predictors in these models, but lack of significance does not rule out smaller effects or effects detectable with more data.

Navigate back to Table 3.

## Table 4 Long description

The table reports likelihood ratio tests from generalized linear models, showing how model fit changes when each predictor is removed for two outcomes: parasite diversity (Poisson) and nematode infection intensity (negative binomial). For parasite diversity in the model with provisioning, removing average human proximity worsens fit (LRT 4.4, p 0.036) and removing rank worsens fit more (LRT 9.5, p 0.009), while provisioning rate (p 0.966) and dog proximity (p 0.109) are not significant. In the parasite diversity model with grooming, removing grooming interactions worsens fit (LRT 6.2, p 0.013), while macaque proximity (p 0.755) and rank (p 0.182) are not significant. For nematode infection intensity in the model with provisioning, only average human proximity is significant (LRT 8.33, p 0.004); provisioning (p 0.238), dog proximity (p 0.835), and rank (p 0.336) are not. In the nematode intensity model with grooming, none of the tested predictors are significant (macaque proximity p 0.113; grooming p 0.968; rank p 0.288). Deviance and AIC values are provided for each reduced model to indicate relative fit, but results reflect single-predictor removals and do not by themselves establish causation.

Navigate back to Table 4.

## Table 5 Long description

Likelihood ratio tests compare full linear regression models with versions that remove one predictor at a time, using changes in residual sum of squares, AIC, and P-values. For dogs, removing provisioning, humans in proximity, human-dog aggression, or rank does not significantly change model fit: P-values range from 0.437 to 0.952, with residual sum of squares around 1.12 to 1.18 and AIC around minus 84 to minus 85. The dog null model has residual sum of squares 1.12 and AIC minus 82. For macaques, the null model has residual sum of squares 4.44 and AIC minus 47. Removing provisioning rate worsens fit and is statistically significant (sum of squares 0.91; residual sum of squares 5.35; AIC minus 44; P 0.018). Removing humans in proximity or rank is not significant for macaques (P 0.141 and 0.125), with residual sum of squares 5.06 and 4.80 and AIC near minus 47. Results indicate provisioning is the only predictor with clear evidence of association with macaque self-scratching in these tests, while none of the tested dog predictors show evidence of contribution.

Navigate back to Table 5.
