# Supplementary material for: Parasite transmission risk at the urban human–dog–macaque interface
Source: Parasitology. 2026 Jun 24;153(8):1038–51. doi: 10.1017/S0031182026102339 (PMC13735960; doi:10.1017/S0031182026102339)
Supplement: Supplementary file 1 [file S0031182026102339sup001.docx]

# **Abstract in Nepali**

;x/Ls/0f / ko{6gn] dfgj–hgfj/ cGt/lqmofnfO{ tLj| agfO/x]sf 5g\, t/ To:tf k|hfltx¿ aLr k/hLjL k|;f/0fsf k|efjx¿ kof{Kt¿kdf a'lemPsf 5}gg\ . o; cWoogdf cTolws ;x/Ls/0f tyf ko{6g k|efljt sf7df8f}F pkTosfsf] :joDe'gfy If]qdf /x]sf dfgj, s's'/ / afFb/df k/hLjLsf] Jofkstf, ljljwtf / ;+qmd0f tLj|tf d"Nof+sg ul/Psf] lyof]] . s's'/ / afFb/x¿sf] cflgaflgsf] cjnf]sg tyf k/hLjL ljZn]if0fdfkm{t dfgjhlgt sf/s / hgfj/x?sf] cflgaflgn] k/hLjL ;+qmd0fdf kfg]{ k|efj k/LIf0f ul/of] . dflg;x¿n] hgfj/x¿;Fusf] glhssf] ;Dks{, pgLx¿nfO{ bfgf–kfgL v'jfpg] / ;/;kmfOsf ;Lldt pkfox¿ h:tf Jojxf/x¿ k|bz{g u/], h;n] k/hLjL ;g]{ hf]lvd a9fpF5 . afFb/x¿df ;a}eGbf pRr k/hLjL ljljwtf kfOof] h'g pgLx¿sf] z/L/–;kmfO ;DaGwL Jojxf/ -u|'ldª_, dflg;;Fusf] glhskg / dfgjåf/f vfB pknAwtf af6 k|efljt lyof] . s's'/x¿df k/hLjLsf] ljljwtf cfˆg} z/L/ sf]6\ofpg] cfgLafgL ;+u cGt/ ;DalGwt lyof] eg]] dfgjdf t'ngfTds ¿kdf sd k/hLjL Jofkstf kfOof] . ;a} k|hfltdf PG6fldaf (*Entamoeba* spp.) sf] klxrfgn] ;femf hf]lvd dfu{ / h'gf]l6s ;DefjgfnfO{ ;+s]t u¥of] . s's'/df k/hLjL ;+qmd0f ;fdflhs jf dfgjhlgt eGbf klg jftfj/0fLo k|b"if0f k|d'v sf/s x'g;Sg] ;+s]t ldNof] . afFb/x¿df ;d"x leqsf] k|e'Tj tx, dfgjåf/f vfB pknAwtf / u|'ldª Jojxf/aLrsf cGt/lqmofn] k/hLjL ljljwtfnfO{ lgwf{/0f u/] . oL kl/0ffdx¿n] ;x/L / ko{6g jftfj/0fn] k|ToIf /f]uhgs ;Dks{ dfq xf]Og, hgfj/x?sf] cflgaflg tyf ;fdflhs ;+oGqåf/f k/f]If ¿kdf klg ;+qmd0f hf]lvdnfO{ k|efljt ug]{ b]vfpF5g\ . dfgj–3/kfn'jf hgfj/–jGohGt' ;x–cl:tTjo'Qm k|0ffnLdf h'gf]l6s hf]lvd Joj:yfkgsf nflu ;x/L kfl/l:yltsL / hgfj/x?sf] cflgaflgsf] PsLs[t b[li6sf]0f ckl/xfo{ b]lvG5 .
