# Supplementary material for: Parasite transmission risk at the urban human–dog–macaque interface
Source: Parasitology. 2026 Jun 24;153(8):1038–51. doi: 10.1017/S0031182026102339 (PMC13735960; doi:10.1017/S0031182026102339)
Supplement: Supplementary file 2 [file S0031182026102339sup002.docx]

**Supplementary materials**

**Table S1.** Ethogram used for each species, dog and macaque respectively, which includes activities (state behaviours) and short-duration behaviours (events).

|  | **Behaviour** | **Definition for dogs** | **Definition for macaques** |
| --- | --- | --- | --- |
| **Activity** | grooming | Dog licks and gently bites without harm another individual (dog/human/macaque) for a prolonged period over 10 seconds | A macaque visually examines, manipulates and cleans the body of another macaque with the fingers |
| **Agonistic behaviours** | Facial Threat | Dog bares their teeth against another individual (dog/human/macaque). This behaviour is often associated with a growl. | A macaque pulls up its eyebrows and scalp, and flattens its ears against the head, and stares at another individual. |
|  | Aggressive stand | Dog stays on its four paws in tense position, not moving, head lowered, teeth showing, hair on the back up and staring at another individual ( dog/human/macaque). This behaviour might be associated with aggressive vocalisation. |  |
|  | Lunge | The aggressor (dog/human/macaque) makes a sudden rapid movement at a short distance (less than 5 meters) towards the victim (dog/human/macaque). In dogs, this behaviour might be associated with aggressive facial threats and vocalisation while in macaques this behaviour might be associated with a slap on the ground. | |
|  | Chase | The aggressor (dog/human/macaque) runs at high speed toward the victim (dog/human/macaque) for **more** than 5 metres. In dogs, this behaviour might be associated with aggressive facial threats and vocalisation. | |
|  | Slap |  | The aggressor (human/macaque) hits or strikes with the hand the victim (human/macaque). |
|  | Bite | The aggressor (dog/macaque) uses their teeth to cut into or through the victim (dog/human/macaque). | |
| **Approaches** | Displacement | The subject (dog/human/macaque) moves towards another individual (dog/human/macaque) who moves away. This movement is done at a slow speed (as compared to chase). | |
|  | Approaching | The subject (dog/human/macaque) approaches another individual (dog/human-macaque) to a distance of less than 2 m and stays within 2 m for at least 3 seconds. | |
|  | Departing | The subject (dog/human/macaque) departs from another individual (dog/human/macaque)’s proximity (2 m) . | |
| **Self-directed behaviours** | Self-scratching | The focal dog uses claws along the skin of its own body in rapid strikes. Counts as one scratching if dog stop scratching for less than 3 seconds | The focal macaque runs its fingers and nails through the skin of its own body in rapid strikes. Counts as one scratching if monkey stops scratching for less than 3 seconds |
| **Human-dog interactions**  **and**  **Human-macaque interactions** | Feeding by hand | A human gives food item by hand; contact between dog/macaque and human. | |
|  | Stealing food | Subject (dog/macaque) takes food from a human without human consent. | |
|  | Throwing food | A human propels food item toward dog/macaque. | |
|  | Touch | A human uses the hand to intentionally make physical contact with dog/macaque without any aggressive intention. | |
|  | Beating | A human hits dog/macaque with its body (e.g., hand, foot) or object (e.g., stick). | |

**Table S2.** Presence or absence of parasite taxa repeatability between the two faecal samples using percentage of agreement, Cohen’s Kapa and PABAK calculations.

|  | **Percentage Agreement (Observed agreement)** | **Cohen’s Kappa** | **PABAK (Prevalence-Adjusted Bias-Adjusted Kappa)** |
| --- | --- | --- | --- |
| *Ascaris* spp. | 94.44 | 0.26 | 0.89 |
| Balantioides/Buxtonella‑like ciliate | 91.11 | 0.75 | 0.82 |
| *Bertiella* spp. | 98.89 | 0 | 0.98 |
| *Blastocystis* spp. | 98.89 | 0 | 0.98 |
| *Chilomastix mesnili* | 98.89 | 0.79 | 0.98 |
| *Dipylidium caninum* | 98.89 | 0.79 | 0.98 |
| *Endolimax* spp. | 97.78 | -0.11 | 0.96 |
| *Entamoeba coli* | 92.22 | 0.33 | 0.84 |
| *Entamoeba* spp. | 90.00 | 0.69 | 0.80 |
| *Enterobius* spp. | 97.78 | -0.11 | 0.96 |
| *Giardia lamblia* | 98.89 | 0 | 0.98 |
| *Iodamoeba buetschlii* | 97.78 | 0.74 | 0.96 |
| *Cystoisospora* spp. | 98.89 | 0 | 0.98 |
| *Physaloptera* spp. | 98.89 | 0.79 | 0.98 |
| Strongylida (indet.) | 86.67 | 0.72 | 0.73 |
| *Strongyloides* spp. | 84.44 | 0.67 | 0.69 |
| *Toxascaris leonina* | 98.89 | 0 | 0.98 |
| *Toxocara canis* | 95.56 | 0.58 | 0.91 |
| *Trichuris* spp. | 96.67 | 0.86 | 0.93 |

**Table S3.** Results of Pearson’s correlation test calculated for each pair of predictor in the macaque data set, run using the R function *Mypairs* in order to assess collinearity between predictors in the GLM model. Numbers represent correlation coefficient *r*. This analysis was run

GR = Grooming rates; MP = Mean number of macaques in proximity; HP = Mean number of humans in proximity; DP = Mean number of dogs in proximity; PR = Provisioning rates; HMA = Human-macaque aggression rates; HMI = Human-macaque interaction rates

**Table S4.** Results of Pearson’s correlation test calculated for each pair of predictor in the dog data set, run using the R function *Mypairs* in order to assess collinearity between predictors in the GLM model. Numbers represent correlation coefficient *r*. This analysis was run

MP = Mean number of macaques in proximity; HP = Mean number of humans in proximity; DP = Mean number of dogs in proximity; PR = Provisioning rates; HDA = Human-dog aggression rates; HDI = Human-dog interaction rates

**Figure S1.** Comparison of total parasite diversity between dogs, macaques and humans. The central black line within each box represents the **median**. The **box edges** indicate the **first (Q1) and third (Q3) quartiles**, capturing the interquartile range (IQR). The **whiskers** extend to the smallest and largest values within 1.5 × IQR from the quartiles. Data points outside this range are plotted as **individual outliers**. The size of the box reflects the spread of the middle 50% of the data. Horizontal lines indicate groups that significantly differ in parasite diversity. *** = p < 0.001.

**Figure S2**. Comparison of nematode density between dogs, macaques and humans. The central black line within each box represents the **median**. The **box edges** indicate the **first (Q1) and third (Q3) quartiles**, capturing the interquartile range (IQR). The **whiskers** extend to the smallest and largest values within 1.5 × IQR from the quartiles. Data points outside this range are plotted as **individual outliers**. The size of the box reflects the spread of the middle 50% of the data. Horizontal lines indicate groups that significantly differ in nematode density. *** = p < 0.001.

**Figure S3a**. Photomicrographs of the protozoan parasites observed during this study; **a**- Cyst of *Balantioides* or Buxtonella-like ciliate, **b**- Trophozoite of *Balantioides* or Buxtonella-like ciliate, **c**- Cyst of *Blastocystis* spp., **d**- Cyst of *Chilomastix mesnili*, **e**- Oocyst of *Cystoisospora* spp.., **f**- Cyst of *Endolimax* spp., **g**- Cyst of *Entamoeba coli*, **h**- Cyst of *Entamoeba* spp., **i**- Cyst of *Giardia* spp., and **j**- Cyst of *Iodamoeba buetschlii*.

**Figure S3b**. Photomicrographs of the helminth parasites observed during this study; a- Egg of *Bertiella* spp., b- Egg of *Dipylidium caninum*, c- Egg of *Ascaris* spp., d- Egg of *Enterobius* sp., e- Egg of *Physaloptera* spp., f- Egg of Strongylida (indet.), g- Egg of *Strongyloides* spp., h- Larva of *Strongyloides* spp., i- Egg of *Toxascaris leonina*, j- Egg of *Toxocara canis*, and k- Egg of *Trichuris* spp.
